# Hypermethylation of the glutathione peroxidase 4 gene promoter is associated with the occurrence of immune tolerance phase in chronic hepatitis B

**DOI:** 10.1186/s12985-024-02346-6

**Published:** 2024-03-21

**Authors:** Xing Su, Zhaohui Wang, Jihui Li, Shuai Gao, Yuchen Fan, Kai Wang

**Affiliations:** 1 Department of Hepatology, Qilu Hospital of Shandong University, Wenhuaxi Road 107#, 250012 Jinan City, Shandong Province China; 2 Hepatology Institute of Shandong University, 250012 Jinan, Shandong China

**Keywords:** Chronic hepatitis B, Glutathione peroxidase 4, STING, DNA methylation, Oxidative stress

## Abstract

**Background:**

Hepatitis B virus (HBV) infection is a public health problem that seriously threatens human health. This study aimed to investigate the clinical significance of glutathione peroxidase 4(GPX4) in the occurrence and development of chronic hepatitis B (CHB).

**Methods:**

A total of 169 participants including 137 patients with CHB and 32 healthy controls (HCs) were recruited. We detected the expression of *GPX4* and stimulator of interferon genes (*STING)* in peripheral blood mononuclear cells (PBMCs) by real-time quantitative polymerase chain reaction (RT-qPCR). The methylation level of *GPX4* gene promoter in PBMCs was detected by TaqMan probe-based quantitative methylation-specific PCR (MethyLight). Enzyme-linked immunosorbent assay (ELISA) was performed to detect the serum levels of GPX4, IFN-β, oxidative stress (OS) related molecules, and pro-inflammatory cytokines.

**Results:**

The expression levels of GPX4 in PBMCs and serum of CHB patients were lower than those of HCs, but the methylation levels of *GPX4* promoter were higher than those of HCs, especially in patients at the immune tolerance phase. *STING* mRNA expression levels in PBMCs and serum IFN-β levels of patients at the immune activation phase and reactivation phase of CHB were higher than those at other clinical phases of CHB and HCs. *GPX4* mRNA expression level and methylation level in PBMCs from patients with CHB had a certain correlation with *STING* and IFN-β expression levels. In addition, the methylation level of the *GPX4* promoter in PBMCs from patients with CHB was correlated with molecules associated with OS and inflammation.

**Conclusions:**

GPX4 may play an important role in the pathogenesis and immune tolerance of CHB, which may provide new ideas for the functional cure of CHB.

**Supplementary Information:**

The online version contains supplementary material available at 10.1186/s12985-024-02346-6.

## Background

Viral hepatitis B is a viral infection that damages the liver and can cause acute or chronic disease. Chronic infection with HBV can progress to liver cirrhosis and hepatocellular carcinoma(HCC), which is a serious threat to human health and global public health [1]. World Health Organization (WHO) estimates that in 2019, 296 million people worldwide were chronically infected with HBV, with 1.5 million new infections and 820,000 deaths each year [2]. In China, an estimated 43.3 million persons remained infected with HBV in 2021 (~ 3.0%) [3]. Therefore, it is of great clinical significance to treat CHB and prevent disease progression. Chronic HBV infection is the result of long-term complex interactions between the host immune system and the virus. According to the levels of hepatitis B surface antigen (HBsAg), hepatitis B e antigen (HBeAg), serum hepatitis B DNA (HBV DNA), and alanine aminotransferase (ALT), the natural course of chronic HBV infection can be roughly divided into five phases: immune tolerance(IT) phase, immune activation(IA) phase, inactive phase [low replication(LR) phase], reactivation(RA) phase and remission phase [4]. Under normal conditions, the host immune system can recognize HBV and produce an anti-HBV response, which is manifested by elevated aminotransferase levels, and liver inflammation of different degrees, and it is possible to obtain HBV DNA clearance and serological transfer of HBeAg through this. While in the immune tolerance phase, the ALT level continues to be normal, the liver has only mild or no inflammatory necrosis, and the host does not produce an immune response against HBV [5]. Therefore, it is of great significance to regulate the immune function of the body and initiate the anti-HBV immune response to break the immune tolerance after HBV infection and clear the virus [6].

The innate immune system is an important part of host immunity, which is composed of physical barriers, phagocytes, cytokines, interferon (IFN) and interferon-stimulating genes (ISGs), and is the first line of defense of the body. Cyclic guanosine monophosphate (GMP)–adenosine monophosphate (AMP) synthase(cGAS) is a DNA recognition receptor located within the cytoplasm. When abnormal DNA is present in the cytoplasm, including exogenous DNA produced by viruses and bacteria, as well as endogenous DNA such as nuclear DNA and mitochondrial DNA, can be recognized and combined by cGAS to synthesize cyclic GMP-AMP (cGAMP) and activate downstream STING [7]. Studies have shown that the cGAS-STING signaling pathway can produce protective immune responses against pathogens with a variety of DNA structures, such as human immunodeficiency virus (HIV), herpes simplex virus 1(HSV-1), human papillomavirus (HPV), and so on [8–10]. STING is an endoplasmic reticulum-associated dimer protein, and its activation plays an important role in the immune response mediated by the cGAS-STING pathway. After activation, STING will undergo spatial conformational changes and transfer from the endoplasmic reticulum (ER) to the Golgi. On the one hand, it recruits and activates TANK-binding kinase 1 (TBK1) and interferon regulatory factor 3 (IRF3) to phosphorylate and transfer from cytoplasm to the nucleus to synthesize interferon. On the other hand, the nuclear factor-κB (NF-κB) dimer is activated to produce inflammatory cytokines including interleukin-1β (IL-1β), interleukin-6 (IL-6) and tumor necrosis factor-α (TNF-α) [7, 11]. STING signaling pathway initiates innate immune response to clear intracellular pathogens by releasing IFN and inflammatory cytokines, and also activates antigen-presenting cells (APC) to present antigens to T cells, thus activating adaptive immune response [7, 12].

GPX4 is a unique enzyme that protects cells against membrane lipid peroxidation and maintains redox homeostasis, by reducing highly reactive lipid hydroperoxides (LOOHs) to nonreactive lipid alcohols [13]. Redox homeostasis is the balance of oxidation and reduction reactions present in all living systems and is an important component of physiological cell homeostasis. Impaired redox homeostasis, such as imbalances in lipid peroxide abundance, is associated with multiple pathological conditions, including viral diseases and cancer [14, 15]. It has been demonstrated that cellular redox homeostasis maintained by GPX4 is necessary for STING activation. GPX4 deficiency enhances cellular lipid peroxidation, thereby specifically inhibiting the cGAS-STING pathway [16]. Therefore, GPX4 deficiency may exist in patients with CHB, thus inhibiting the immune response of the cGAS-STING pathway to HBV and forming an immune tolerance state.

In this study, we primarily used MethyLight to detect the methylation status of *GPX4* promoter in patients with CHB and HCs. In addition, we detected GPX4, STING and IFN-β expression levels to evaluate the potential relationship between *GPX4* promoter methylation and expression levels of cGAS-STING pathway-related molecules in patients with CHB. The aim is to elucidate the potential clinical significance of GPX4 in the occurrence and development of CHB.

## Materials and methods

### Subjects

A total of 169 participants were recruited from January 2022 through February 2023 in the Department of Hepatology, Qilu Hospital of Shandong University including 137 patients with CHB and 32 HCs. All patients with CHB were selected and clinically staged into the IT phase, IA phase, LR phase, and RA phase based on 2018 AASLD Practice Guidelines [17].Healthy volunteers served as normal controls with negative viral hepatitis tests, normal ALT/AST levels, and no evidence of other liver or malignant diseases. This research was reviewed and approved by the Medical Ethical Committee of Qilu Hospital of Shandong University and was conducted according to the Declaration of Helsinki. All participants signed informed consent after understanding the experimental process and required specimens.

### Serum collection and PBMCs isolation

Peripheral venous blood(5 ml) was collected from each subject into an EDTA-containing tube following an 8-hour fast and whole blood was also collected in additional serum separator tubes (SST) for serum isolation. Following centrifugation, the serum was isolated and PBMCs were obtained through Ficoll-Paque Plus (GE Healthcare, Uppsala, Sweden) density gradient centrifugation. Then the obtained serum and PBMCs were either used immediately for downstream experiments or preserved at a temperature of − 80℃ until required.

### DNA extraction and MethyLight

Genomic DNA was extracted from PBMCs using TRIzol Reagent (Invitrogen, Carlsbad, CA, USA). EZ DNA Methylation-Gold kit (Zymoresearch, Orange, CA, USA) was used for DNA bisulfite modification. MethyLight was performed using the EpiTect MethyLight PCR + ROX Vial Kit (QIAGEN, Hilden, Germany). Two sets of primers and probes designed specifically for bisulfite-converted DNA were used: an experimental set for the *GPX4* gene and a reference set for the β-actin gene, which served as a normalization control. We used the website (https://www.ncbi.nlm.nih.gov/) to delineate the promoter of *GPX4* and another website (https://www.urogene.org/methprimer/) for sequence transformation. Then, the Oligo7 (OLIGO 1267 Vondelpark ColoradoSprings, CO 80,907, USA) was used for the sequence design of probes and primers. The genome coordinates of *GPX4* are hg38, chr19: 1,103,994–1,106,779. We selected the upstream 2,000 bp region of its transcription start site (TSS) as the promoter region. Then, we found one CpG island (from 1,678 bp to 1,945 bp) through the website (https://www.urogene.org/methprimer/) [18]. So, the primers and probes were designed at the CpG island region (Supplementary Fig. S1). The specific primers and probe sequences for *GPX4* and β-actin gene promoters are listed in Table 1.

**Table 1:** Sequences of used primers and probes Probe modification: 5′6-FAM and 3′BHQ1

	Gene	Forward primer sequence (5′ to 3′)	Reverse primer sequence (5′ to 3′)	Probe sequence (5′ to 3′)
RT-qPCR	GPX4	AAGTAAACTACACTCAGCTCGTC	AAACCACACTCAGCGTATCGG	
	STING	TAACCTGAGTATGGCTGACCCCAA	CTGTAAACCCGATCCTTGATGC	
	β-actin	CATGTACGTTGCTATCCAGGC	CTCCTTAATGTCACGCACGAT	
MethyLight	GPX4	TCGACGGGTATATGGTTAATTTGGAT	GTTAATAACGATACACACGAAACCCCTA	CCAAACGAACGCCCACCGAT
	β-actin	TGGTGATGGAGGAGGTTTAGTAAGT	AACCAATAAAACCTACTCCTCCCTTAAA	ACCACCACCCAACACACAATAACAAACACA

We used a total volume of 10 µl following the standard protocol provided by the manufacturer: 95℃ for 15 min, followed by 45 cycles of 95℃ for 15 s and 60℃ for 1 min. The percentage of the methylation reference value (PMR) indicates MethyLight data. PMR = 100% x 2 exp. [Delta Ct (target gene-control gene) Sample- Delta Ct (target gene-control gene) M.SssI-Reference] [19].

### RNA extraction and RT-qPCR

Total RNA was extracted from PBMCs using TRIzol Reagent (Invitrogen, Carlsbad, CA, USA) and cDNA was then immediately synthesized using the First-Strand cDNA Synthesis Kit (Fermentas, Vilnius, Lithuania) according to the manufacturer’s instructions. Complementary DNA (cDNA) was used as a template immediately for RT-qPCR. The 10 µl reaction system with the condition of denaturation at 95℃for 30s, followed by 45 cycles of 95℃ for 5s, 60℃ for 30s and 72℃ for 30s. The sequences of specific primers for *GPX4*, *STING* and β-actin were all described in Table 1. The relative expression of *GPX4* and *STING* was calculated using the 2 − ΔΔCT method. All amplification reactions were performed in triplicate.

### ELISA

Serum cytokine levels were measured using the Human Immunoassay Valukine ELISA Kits for GPX4, IFN-β, reactive oxygen species (ROS), superoxide dismutase (SOD), malonaldehyde (MDA), TNF-α, IL-1β and IL-6 (Lengton Bioscience Co, Shanghai, China). These kits employ a competitive method to detect samples’ content, and absorbance was measured at 450 nm according to the manufacturer’s protocol. All samples were measured in triplicate.

### Statistical analysis

Statistical analyses were performed using SPSS version 26.0 statistical software (SPSS Inc., Chicago, IL, USA). Quantitative variables are expressed as median (centile 25; centile 75). Categorical variables were expressed as numbers (%). Mann–Whitney U test, Kruskal-Wallis Test and Dunn’s test were used to compare the quantitative variables. The chi-square test was used to compare the categorical variables. The Spearman’s rank correlation test was used to analyze the relationship between *GPX4* promoter methylation level and quantitative clinical data as well as *GPX4* mRNA expression level, *STING* mRNA expression level, serum GPX4, IFN-β, ROS, SOD, MDA, TNF-α, IL-1β and IL-6 levels. The gender and all other statistical analyses were dichotomous variables. All statistical analyses were 2-sided, and *P* value < 0.05 was considered statistically significant.

## Results

### General characteristics of the study populations

In this study, a total of 169 participants were enrolled, including 46 patients with CHB in the immune tolerance phase, 50 patients with CHB in the immune activation phase, 20 patients with CHB in the inactive low replication phase, 21 patients with CHB in reactivation phase and 32 HCs. The demographic characteristics, clinical manifestations, and laboratory measurements of the subjects are shown in Table 2.

**Table 2:** General clinical characteristics of the patients

Variables	IT(*n*=46)	IA(*n*=50)	LR(*n*=20)	RA(*n*=21)	HC(*n*=32)	P value
Male, n (%)	22(47.83)	37(74.00)	9(45.00)	13(61.90)	18(56.25)	0.067 ^c^
Age (years)	35(31-46)	39(34-49)	40(31-55)	44(36-49)	48(32-58)	0.053 ^b^
HbsAg(IU/ml)	33725.95(6390.21-54364.65)	10819.65(4075.77-21753.43)	3679.90(1095.77-7375.08)	3050.29(1684.47-8662.55)	NA	<0.001 ^b^
HBeAg(IU/ml)	1394.59(541.24-1588.91)	880.70(262.23-1436.98)	NA	NA	NA	<0.001 ^a^
log10[HBV-DNA]	7.97(5.25-8.40)	6.92(5.49-8.06)	3.45(3.08-3.96)	4.79(3.47-5.81)	NA	<0.001 ^b^
ALT(U/L)	27.50(17.75-35.00)	103.00(66.25-225.50)	20.00(15.00-27.75)	74.00(25.50-154.50)	15.50(11.50-23.50)	<0.001 ^b^
AST(U/L)	21.50(18.00-27.25)	65.00(44.75-114.25)	20.00(18.00-24.75)	41.00(22.50-99.50)	19.00(15.50-22.00)	<0.001 ^b^
AKP(U/L)	77.00(61.75-94.25)	90.50(76.00-104.50)	75.50(62.75-97.50)	88.00(66.50-98.00)	74.50(57.50-84.00)	0.029 ^b^
GGT(U/L)	19.00(12.00-31.00)	38.50(27.25-94.50)	16.50(12.25-25.25)	30.00(19.50-61.50)	21.00(13.25-27.75)	<0.001 ^b^
TBIL(µmol/L)	9.45(7.50-14.10)	13.70(9.30-16.98)	10.45(8.03-14.33)	12.30(9.15-18.10)	9.70(8.15-14.33)	0.007 ^b^
ALB(g/L)	46.25(44.20-48.83)	45.60(43.15-48.83)	47.80(46.10-49.93)	48.20(45.45-50.30)	47.15(46.05-49.03)	0.064 ^b^
Cr(µmol/L)	72.00(54.00-79.00)	69.00(60.00-76.50)	65.00(57.00-78.00)	71.50(56.25-78.75)	69.50(64.25-79.00)	0.903 ^b^
PLT(10^9/L)	218.00(182.75-252.00)	192.50(154.25-232.25)	220.00(183.75-235.00)	185.00(151.50-224.00)	228.50(200.25-272.75)	0.005 ^b^
PTA(%)	119.00(109.00-122.75)	103.50(88.50-107.75)	115.00(110.50-122.00)	102.00(92.00-109.00)	110.00(103.00-119.00)	0.007 ^b^
INR	0.90(0.89-0.95)	0.98(0.96-1.08)	0.92(0.89-0.94)	0.99(0.95-1.06)	0.94(0.90-0.98)	<0.001 ^b^
AFP(ng/ml)	2.41(1.81-3.60)	5.68(2.83-15.89)	2.06(1.48-3.16)	2.82(1.92-4.50)	2.90(2.46-4.75)	<0.001 ^b^

Quantitative variables were expressed as medians (25th, 75th percentage)

Qualitative variables were expressed as number (percentage)

a Mann–Whitney U test. b Kruskal–Wallis H test. c chi-square test

### The expression levels of GPX4 are decreased in different phases of CHB

To investigate the potential involvement of GPX4, we initially examined GPX4 expression levels in patients with CHB in different phases and HCs. First, we analyzed the relative mRNA level of *GPX4* in PBMCs from IT, IA, LR, RA and HCs(Fig. 2a). Compared to HCs, the relative mRNA level of *GPX4* was significantly lower in IT(*P* < 0.0001), IA(*P* < 0.0001), LR (*P* < 0.0001) and RA(*P* = 0.0092) respectively. And the relative mRNA level of *GPX4* was significantly lower in IT than in IA(*P* = 0.0361) and RA(*P* = 0.0254). Next, we analyzed the methylation status of the *GPX4* promoter expressed as PMR in PBMCs from IT, IA, LR, RA and HCs (Fig. 2b). Compared to HCs, the *GPX4* promoter methylation level was significantly higher in IT (*P* < 0.0001), IA (*P* = 0.0002), LR (*P* = 0.0284) and RA(*P* = 0.0475) respectively. And the *GPX4* promoter methylation level was significantly higher in IT than in IA(*P* = 0.0436) and RA(*P* = 0.0449). Besides, we analyzed the serum GPX4 level from IT, IA, LR, RA and HCs (Fig. 2c). Compared to HCs, the serum GPX4 level was significantly lower in IT (*P* < 0.0001), IA (*P* < 0.0001), LR (*P* < 0.0001) and RA (*P* = 0.0075) respectively. And the serum GPX4 level was significantly lower in IT than in IA (*P* = 0.0012) and RA(*P* = 0.0105).

**Fig. 1:**
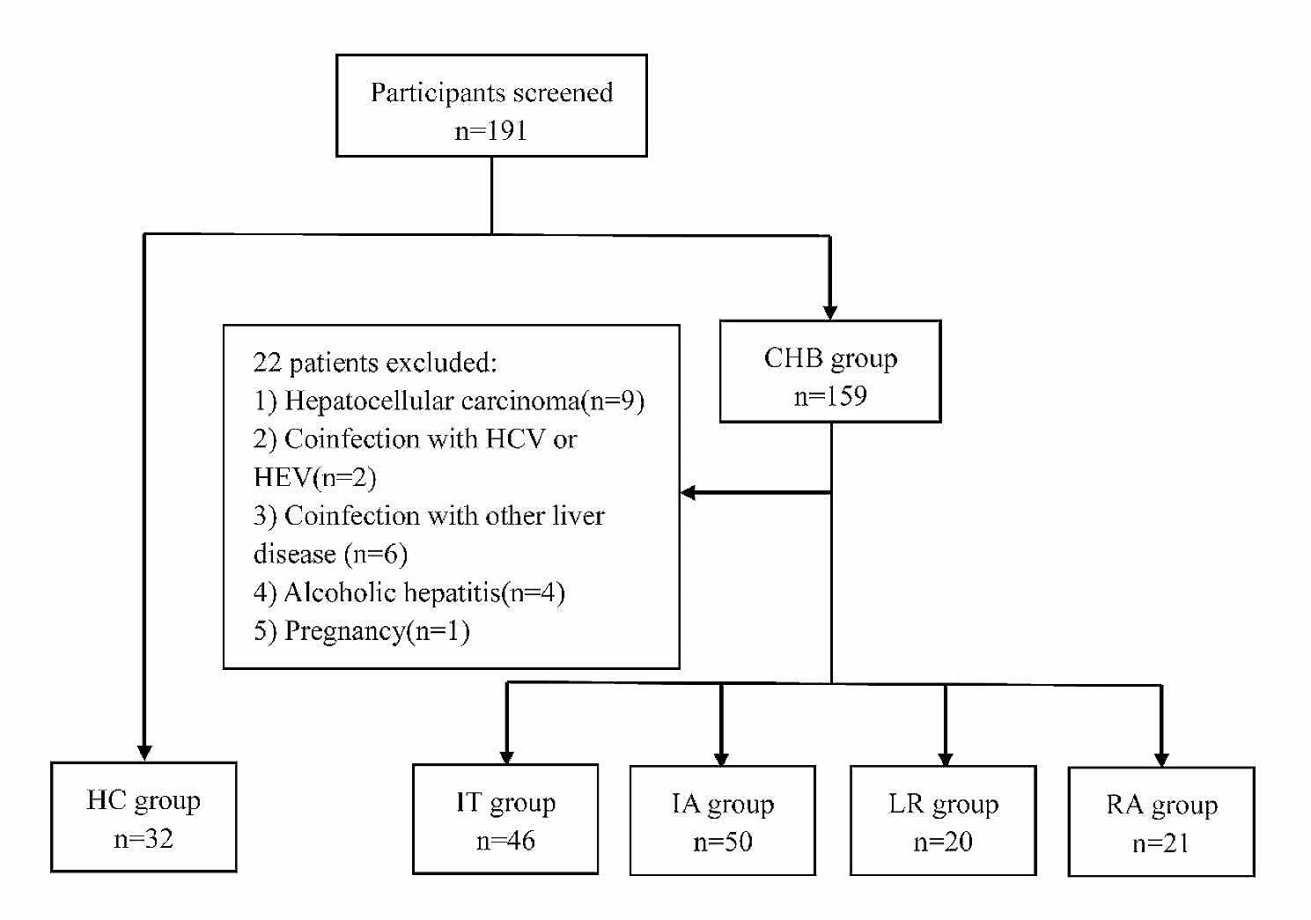
The exclusion criteria of subjects

**Fig. 2:**
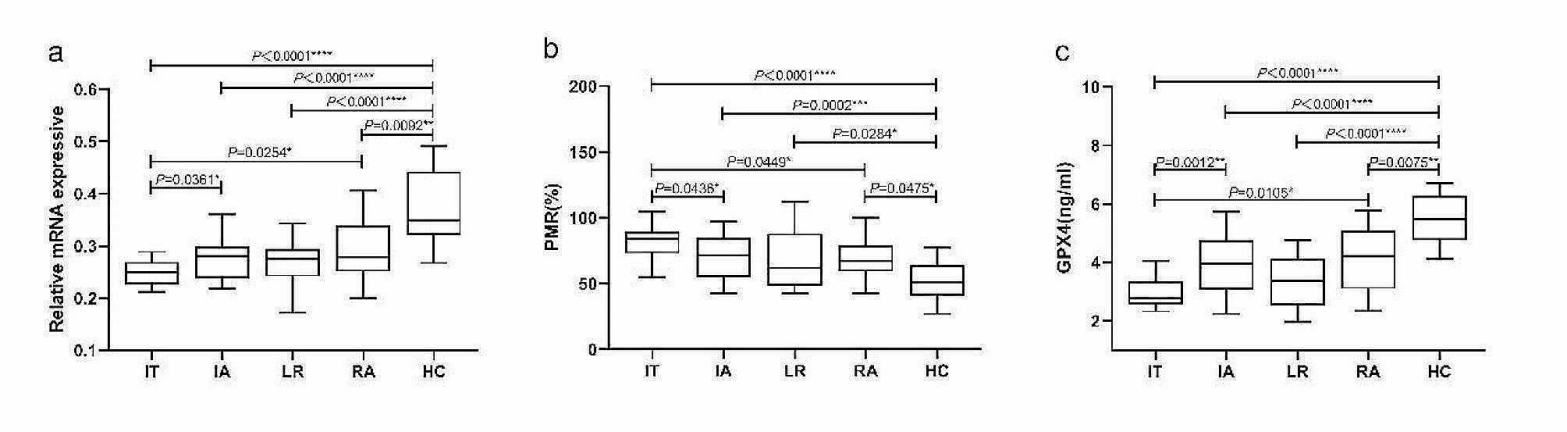
The expression patterns of GPX4 in different groups. (**a)** Relative mRNA level of *GPX4* in PBMCs from IT, IA, LR, RA and HCs. Relative mRNA level of *GPX4* was significantly lower in IT(*P* < 0.0001), IA(*P* < 0.0001), LR(*P* < 0.0001) and RA(*P* = 0.0092) than in HCs, respectively, by using the Kruskal-Wallis Test and Dunn’s test. (**b)***GPX4* promoter methylation level in PBMCs from IT, IA, LR, RA and HCs. *GPX4* promoter methylation level was significantly higher in IT(*P* < 0.0001), IA(*P* = 0.0002), LR(*P* = 0.0284) and RA(*P* = 0.0475) than in HCs, respectively, by using the Kruskal-Wallis Test and Dunn’s test. (**c)** Serum GPX4 level from IT, IA, LR, RA and HCs. Serum GPX4 level was significantly lower in IT(*P* < 0.0001), IA(*P* < 0.0001), LR(*P* < 0.0001) and RA(*P* = 0.0075) than in HCs, respectively, by using the Kruskal-Wallis Test and Dunn’s test. ns, *P*>0.05; *, *P* ≤ 0.05; **, *P* ≤ 0.01; ***, *P* ≤ 0.001; ****, *P* ≤ 0.0001

**Fig. 4:**
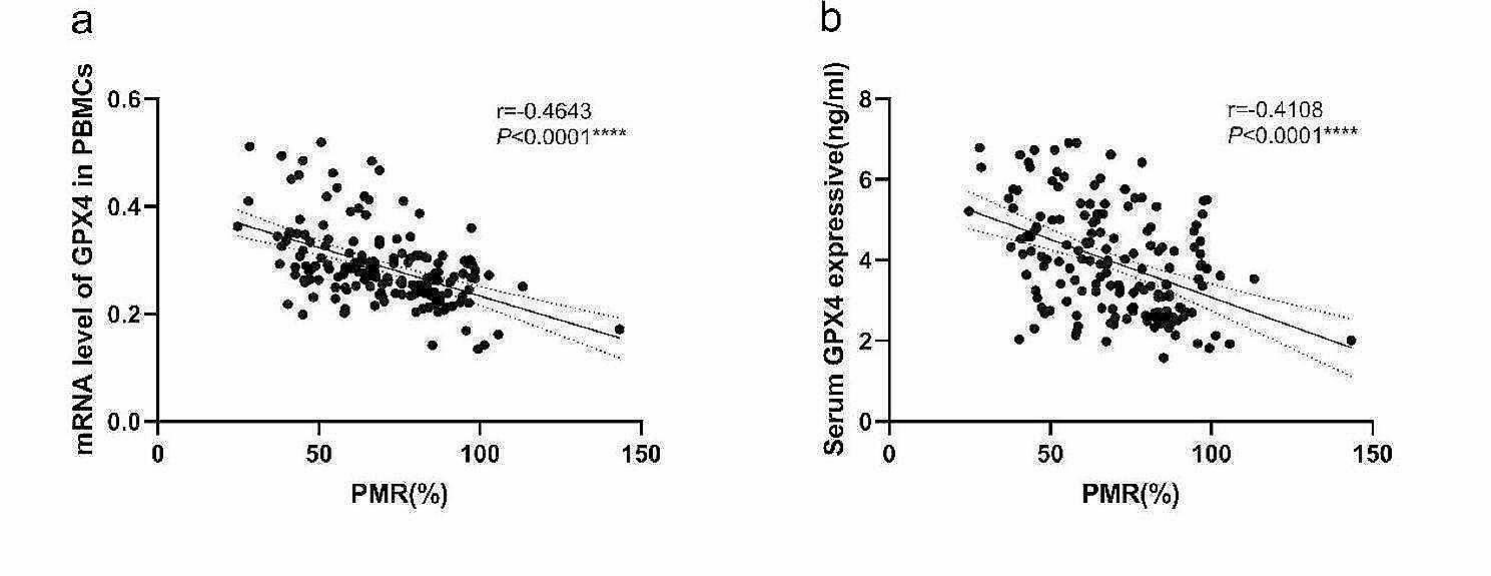
The associations between *GPX4* promoter methylation level and mRNA level in PBMCs, and GPX4 expressive in serum. Significant correlation was observed between the PMR value of *GPX4* promoter and mRNA level in PBMCs (**a** Spearman’s *r* = − 0.4613, *P*<0.0001), and GPX4 expressive in serum (**b** Spearman’s *r* = − 0.4108, *P*<0.0001), ns, *P*>0.05; *, *P* ≤ 0.05; **, *P* ≤ 0.01; ***, *P* ≤ 0.001; ****, *P* ≤ 0.0001

The relationships between *GPX4* methylation levels and *GPX4* mRNA levels in PBMCs and between *GPX4* methylation levels and serum GPX4 expression levels in patients with CHB was further analyzed by using Spearman rank correlation analysis. We found the PMR value of *GPX4* was significantly negatively correlated with the mRNA level of *GPX4* in PBMCs (Spearman’s *r* = − 0.4613, *P*<0.0001) and serum GPX4 expression level (Spearman’s *r* = − 0.4108, *P*<0.0001) as Fig. 4.

### The expression levels of STING and IFN-β are different in HCs and patients in different phases of CHB

**Fig. 3:**
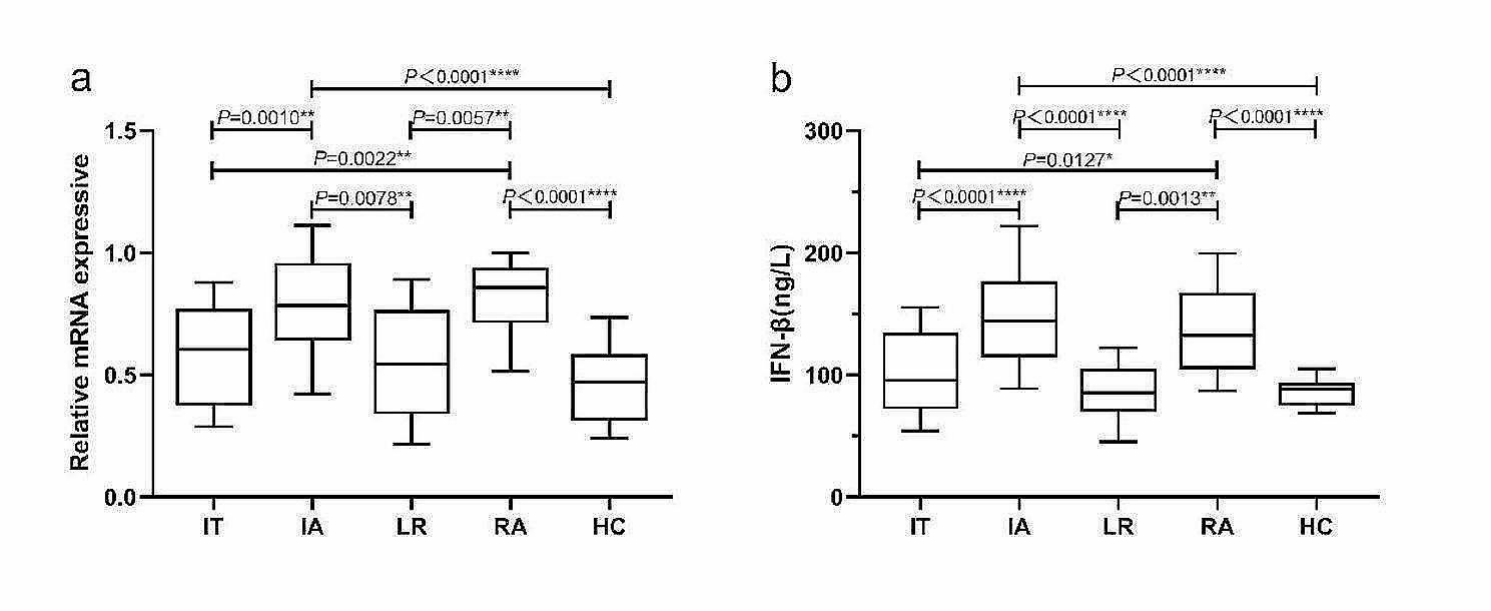
The expression levels of cGAS-STING pathway-related molecules in different groups. (**a)** Relative mRNA level of *STING* in PBMCs from IT, IA, LR, RA and HCs. Relative mRNA level of *STING* was significantly lower in IT(*P* = 0.0010), LR(*P* = 0.0078) and HC(*P* < 0.0001) than in IA and lower in IT(*P* = 0.0022), LR(*P* = 0.0057) and HC(*P* < 0.0001) than in RA, respectively, by using the Kruskal-Wallis Test and Dunn’s test. (**b)** Serum IFN-β level from IT, IA, LR, RA and HCs. Serum IFN-β level was significantly lower in IT(*P* < 0.0001), LR(*P* < 0.0001) and HC(*P* < 0.0001) than in IA and lower in IT(*P* = 0.0127), LR(*P* = 0.0013) and HC(*P* < 0.0001) than in RA, respectively, by using the Kruskal-Wallis Test and Dunn’s test. ns, *P*>0.05; *, *P* ≤ 0.05; **, *P* ≤ 0.01; ***, *P* ≤ 0.001; ****, *P* ≤ 0.0001

**Fig. 5:**
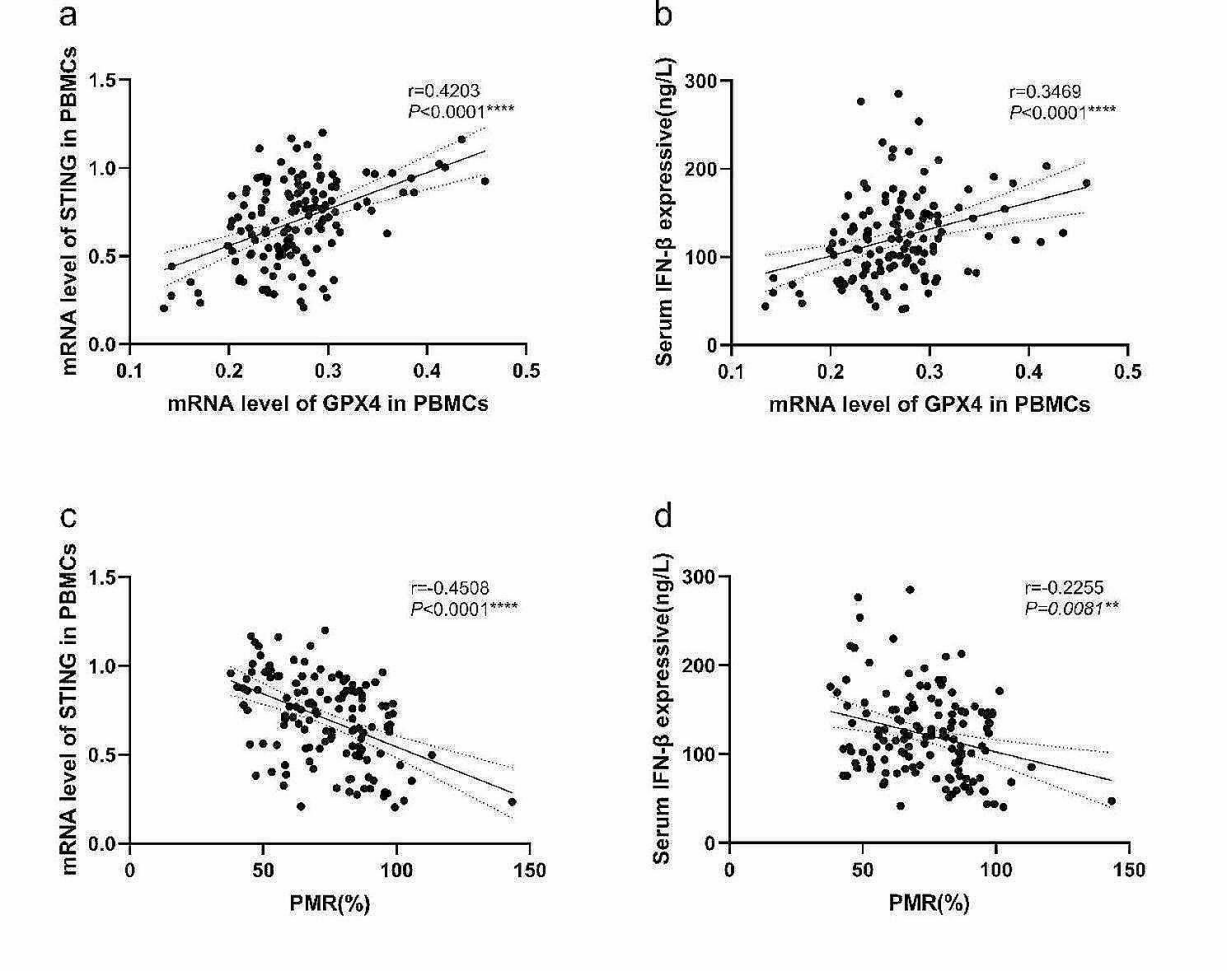
The associations between mRNA level and methylation level of *GPX4* in PBMCs and mRNA level of *STING* in PBMCs, and IFN-β expressive in serum. Significant correlation was observed between the mRNA level of *GPX4* and mRNA level of *STING* in PBMCs (**a** Spearman’s *r* = 0.4203, *P*<0.0001), and IFN-β expressive in serum (**b** Spearman’s *r* = 0.3469, *P*<0.0001). And significant correlation was observed between the PMR value of *GPX4* promoter and mRNA level of *STING* in PBMCs (**c** Spearman’s *r* = − 0.4508, *P*<0.0001), and IFN-β serum expression level (**d** Spearman’s *r* = − 0.2255, *P* = 0.0081),ns, *P*>0.05; *, *P* ≤ 0.05; **, *P* ≤ 0.01; ***, *P* ≤ 0.001; ****, *P* ≤ 0.0001

To explore the role of the cGAS-STING signaling pathway in the development of CHB and its relationship with GPX4, we examined the mRNA expression level of *STING* and serum IFN-β level in HCs and patients in different phases of CHB. The results are shown in Fig. 3. As shown in the figure, the relative mRNA level of *STING* was significantly lower in IT(*P* = 0.0010), LR(*P* = 0.0078) and HC(*P* < 0.0001) than in IA and lower in IT(*P* = 0.0022), LR(*P* = 0.0057) and HC(*P* < 0.0001) than in RA, respectively(Fig. 3a). And the serum IFN-β level was significantly lower in IT(*P* < 0.0001), LR(*P* < 0.0001) and HC(*P* < 0.0001) than in IA and lower in IT(*P* = 0.0127), LR(*P* = 0.0013) and HC(*P* < 0.0001) than in RA, respectively(Fig. 3b). Besides, the relationship between the mRNA level of *GPX4* in PBMCs and *GPX4* methylation level with mRNA level of *STING* in PBMCs and serum IFN-β expression level in patients with CHB was further analyzed by using Spearman rank correlation analysis. We found the mRNA level of *GPX4* in PBMCs was significantly positively correlated with mRNA level of *STING* in PBMCs(Spearman’s *r* = 0.4203, *P*<0.0001, Fig. 5a) and serum IFN-β expression level (Spearman’s *r* = 0.3469, *P*<0.0001, Fig. 5b), and the PMR value of *GPX4* was significantly negatively correlated with mRNA level of *STING* in PBMCs (Spearman’s *r* = − 0.4508, *P*<0.0001, Fig. 5c) and serum IFN-β expression level (Spearman’s *r* = − 0.2255, *P* = 0.0081, Fig. 5d).

### Associations between the *GPX4* promoter methylation level and the serum expression levels of related cytokines in patients with CHB

**Fig. 6:**
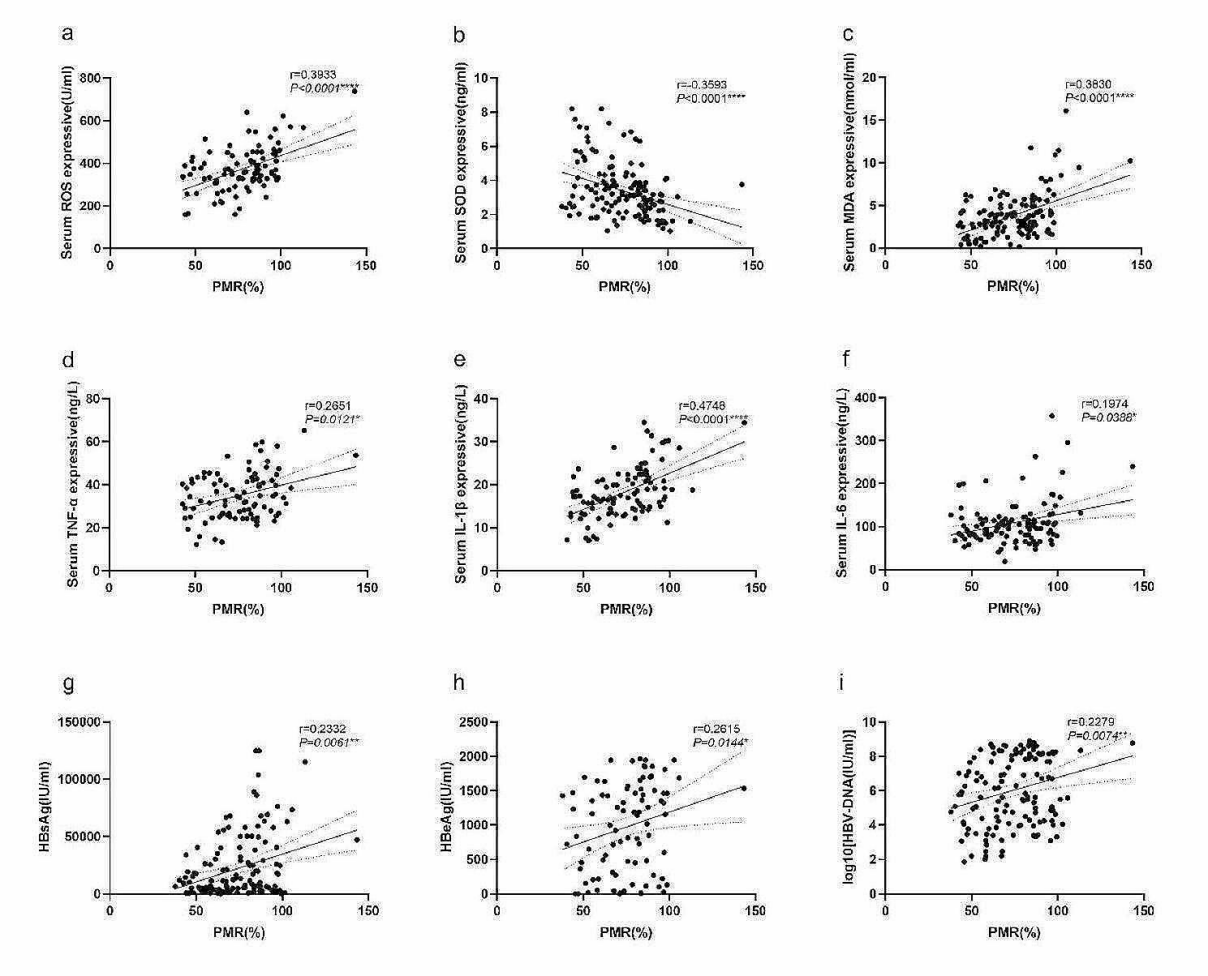
The associations between *GPX4* promoter methylation level and related cytokines serum expression levels, and hepatitis B virus load. Significant correlation was observed between the PMR value of the *GPX4* promoter and ROS serum expression level (**a** Spearman’s *r* = 0.3933, *P*<0.0001), and SOD serum expression level (**b** Spearman’s *r* = -0.3593, *P*<0.0001), and MDA serum expression level (**c** Spearman’s *r* = 0.3830, *P*<0.0001), and TNF-α serum expression level (**d** Spearman’s *r* = 0.2651, *P* = 0.0121), and IL-1β serum expression level (**e** Spearman’s *r* = 0.4748, *P*<0.0001), and IL-6 serum expression level (**f** Spearman’s *r* = 0.1974, *P* = 0.0388), and HBsAg level (**g** Spearman’s *r* = 0.2332, *P* = 0.0061), and HBeAg level (**h** Spearman’s *r* = 0.2615, *P* = 0.0144), and HBV DNA load (**i** Spearman’s *r* = 0.2279, *P* = 0.0074), ns, *P*>0.05; *, *P* ≤ 0.05; **, *P* ≤ 0.01; ***, *P* ≤ 0.001; ****, *P* ≤ 0.0001

To further explore the mechanism(s) underlying the down-regulation of GPX4 during different phases of CHB, we examined cytokines associated with OS, antioxidant stress, and inflammation serum expression levels in patients with CHB by using ELISA. Then the relationships between *GPX4* methylation level and them were further analyzed by using the Spearman rank correlation test and the results are shown in Fig. 6. As shown in the figure, the PMR value of *GPX4* was significantly positively correlated with ROS serum expression level (Spearman’s *r* = 0.3933, *P*<0.0001, Fig. 6a), MDA serum expression level (Spearman’s *r* = 0.3830, *P*<0.0001, Fig. 6c), TNF-α serum expression level (Spearman’s *r* = 0.2651, *P* = 0.0121, Fig. 6d), IL-1β serum expression level (Spearman’s *r* = 0.4748, *P*<0.0001, Fig. 6e), and IL-6 serum expression level (Spearman’s *r* = 0.1974, *P* = 0.0388, Fig. 6f). And, the PMR value of *GPX4* was significantly negatively correlated with SOD serum expression level (Spearman’s *r* = -0.3593, *P*<0.0001, Fig. 6b).

### The *GPX4* promoter methylation level is correlated with HBV load in patients with CHB

Last, we determined whether or not *GPX4* methylation is associated with hepatitis B virus infection. We found the PMR value of *GPX4* was significantly positively correlated with HBsAg level (Spearman’s *r* = 0.2332, *P* = 0.0061, Fig. 6g), HBeAg level (Spearman’s *r* = 0.2615, *P* = 0.0144, Fig. 6h), and HBV DNA load (Spearman’s *r* = 0.2279, *P* = 0.0074, Fig. 6i) of CHB patients.

## Discussion

Chronic HBV infection can lead to intrahepatic inflammation, and the repeated cycle of liver inflammatory injury and repair results in the development of liver fibrosis, irreversible cirrhosis, and even HCC [1]. Effective antiviral therapy has significant implications for improving patient prognosis as it can inhibit HBV replication, normalize liver enzyme levels, reduce liver inflammation, reverse fibrosis progression, and reduce the risk of liver cancer [4]. However, the current status of HBV diagnosis and treatment is not optimistic,and achieving the WHO’s goal of eliminating viral hepatitis by 2030 remains a challenging task. Currently, “functional cure” is considered the ultimate treatment endpoint for chronic HBV infection. This refers to sustained loss of HBsAg along with negative results on HBV DNA tests, sustained normal transaminase levels, and improvement in liver histological conditions after antiviral treatment [20]. Approved treatment options for CHB include nucleos(t)ide analogues and long-acting interferon. Nucleoside drugs are widely used due to their ability to inhibit reverse transcriptase activity effectively and suppress HBV replication while being well-tolerated. However, achieving clearance of HBsAg is difficult with a very low probability (only 0.5%) of attaining a functional cure [21]; most patients require long-term or lifelong medication to continuously suppress viral replication [22]. Additionally, the long-term use of nucleoside drugs carries the risk of drug resistance. Different from nucleos(t)ide analogues, long-acting interferon treatment time is fixed, usually 48 weeks, and HBsAg clearance rate after treatment can reach 3–5% [23, 24], although it improves the HBsAg clearance rate, the side effects of interferon treatment are more, so it is rarely used alone in clinical practice. In general, although the existing antiviral treatment can bring benefits to patients, overall it is still difficult to achieve HBsAg clearance, and the functional cure for HBV still does not yet meet the clinical needs [25, 26]. Based on the existing antiviral drugs, constantly searching for new therapeutic targets and developing new treatment strategies are expected to achieve the goal of eliminating HBV.

To achieve a functional cure for HBV, a combination of drugs with distinct mechanisms is imperative [27]. In terms of immunotherapy, overcoming immune tolerance following HBV infection and stimulating the body to reestablish an anti-HBV immune response present urgent scientific challenges [28]. PPR agonists have exhibited remarkable efficacy in tumor treatment, thus holding the potential for playing a pivotal role in antiviral therapy, particularly against HBV. At present, in the field of CHB treatment, pattern recognition agonists for different targets, including Toll-like receptor agonists, and RIG − 1 agonists, now has entered the stage of HBV drug clinical trials and achieved good results. Activating the body’s natural immune response is likely to achieve functional cure of HBV [27, 29]. However, it is also necessary to be alert that the innate immune response is usually not targeted, which may cause the “cytokine storm” and related adverse reactions [30]. Additionally, various STING agonists are under investigation. Existing research indicates that compared to Toll-like receptor agonists, STING agonists mainly induce immune cells to synthesize IFN-β, and produce lower levels of inflammatory cytokines such as interleukins, which suggests that STING agonists have less risk of inflammatory factor storm and better safety when inducing anti-HBV response [31]. In the mice model of chronic hepatitis with persistent infection of HBV preplasmid-induced recombinant cccDNA (rcccDNA), other researchers have discovered that activation of the STING signaling pathway can inhibit HBV replication by inhibiting ccc DNA-mediated transcription in hepatocytes and epigenetic modifications involved in HBV replication while also reducing liver tissue damage and fibrosis levels [32]. Overall, STING signaling pathways play a significant role in the chronic HBV infection process by disrupting immune tolerance.

. Cellular homeostasis can regulate the activation of the cGAS-STING signaling pathway. For example, cellular manganese (Mn^2+^) ions enhance the sensitivity of cGAS to double-stranded DNA (dsDNA), thereby promoting cGAMP production [33]. Intracellular zinc ion (Zn^2+^) promotes the phase separation of cGAS and DNA binding, which is necessary for maintaining the activation of the cGAS-STING signaling pathway [34]. Redox homeostasis is an important component of cellular homeostasis and a physiological self-correction mechanism to cope with different stress microenvironments such as viral infection and tumors. GPX4 is a kind of glutathione (GSH) and selenium-dependent glutathione peroxidase, a key regulator to the ferroptosis regulation pathway, by scavenging free radicals, participating in the hydrolysis of lipid peroxides, which protect cells from accumulation of lipid peroxides, reduce OS damage, is an antioxidant molecule [35]. Studies have shown that GPX4 is involved in a variety of biological functions, including neuronal loss, autophagy, cell repair, inflammation, ferroptosis, apoptosis and OS [36]. Indirect or direct inhibition of GPX4 leads to insufficient lipid peroxides clearance, accumulation of intracellular lipid peroxides, OS damage and ferroptosis. That is, impaired GPX4 activity is generally positively correlated with increased OS and regulatory cell death (RCD) in disease [37].

Studies have demonstrated the involvement of GPX4 in the pathogenesis and progression of various diseases, including inflammatory disorders, autoimmune conditions, neurodegenerative ailments, ischemia-reperfusion(I/R) injury, tumors, and so on. Notably, GPX4 expression in myeloid-like cells, particularly macrophages, serves to restrict CASP1-CASP11-mediated inflammasome activation and GSDMD-dependent pyroptosis. This mechanism effectively prevents cytokine storm and septic death during bacterial infections [38]. Additionally, GPX4 inhibits the inflammatory NF-κB pathway activation and the production of proinflammatory mediators (e.g., IL1B and PTGS2/COX2) across diverse experimental models, such as angiogenesis and hair follicle development [39, 40]. A mouse model with conditional knockout of *GPX4* in T cells displays an accumulated membrane lipid peroxide phenotype, resulting in an intrinsic defect in protection against acute lymphocytic choriomeningitis virus and Leishmania infection [41]. GPX4 deficiency in the pancreas or small intestine can lead to pancreatitis [42] and inflammatory bowel disease [43]. Furthermore, *GPX4* ablation in neutrophils exacerbates the development of autoimmune diseases, such as systemic lupus erythematosus, by inducing ferroptosis. Collectively, these conditional *GPX4* depletion models suggest a broad regulatory role for GPX4 in the prevention of inflammatory and autoimmune diseases. In the nervous system, GPX4 promotes postnatal neuronal differentiation in mice [44]. Overexpression of GPX4 can prevent I/R injury in some tissues by limiting lipid peroxidation. Loss or inhibition of GPX4 expression accelerates I/R injury. For example, down-regulation of GPX4 expression by Mir135b-3p promotes I/R injury in cardiomyocytes [45]. GPX4 plays a dual role in tumorigenesis and progression. Lack of GPX4 causes cell death and tissue damage. On the one hand, chronic inflammation may occur, leading to DAMP-mediated macrophage polarization in the tumor microenvironment and promoting tumor growth. On the other hand, acute cell death caused by *GPX4* depletion can eliminate tumor cells, release DAMPs, and activate antitumor immunity. Numerous studies have shown that GPX4 inhibitors enhance sensitivity to chemotherapy, radiotherapy, and immunotherapy by inducing ferroptosis [46]. For example, inhibition of GPX4 to enhance ferroptosis can increase the sensitivity of HCC to sorafenib [47], Epstein-Barr virus(EBV)-infected nasopharyngeal carcinoma to platinum [48], etc. But, because GPX4 is widely expressed in anti-tumor immune cells such as T cells, dendritic cells, and neutrophils and in immunosuppressive cells such as Tregs, non-selective targeting of GPX4 in preclinical cancer therapy may lead to immune side effects that suppress anti-tumor immunity.

Of course, GPX4 is also involved in the occurrence and development of some liver diseases. One study found that GPX4 is critical for hepatocyte survival and proper liver function [49]. Targeting GPX4 may become a new way to treat a variety of liver diseases. For instance, inhibiting GPX4 activity induces ferroptosis in HCC cells [47]; targeting GPX4 can mitigate ferroptosis to treat metabolic-associated fatty liver disease [50]. Activation of GPX4 by Maresin1 (MaR1) alleviates liver injury while reducing the expression of ROS, MDA, and inflammatory factors [51]. However, there is no relevant study on GPX4 in CHB. Considering that GPX4 is widely expressed in immune cells and is the first line of defense of the immune system against inflammation, we chose PBMCs for detection. Moreover, some related studies have identified impaired expression of the *GPX4* gene in PBMCs from breast cancer patients which could serve as a biomarker indicating an increased risk for breast cancer [52], indicating that the expression of GPX4 in PBMCs is indeed related to some particular disease.

DNA methylation, a chemical modification of DNA, is a type of epigenetics. DNA methylation is the acquisition of methyl groups by DNA methyltransferases (DNMTs), which in turn affects gene expression without changing the DNA sequence. DNA methylation can inhibit gene expression, leading to lower gene expression levels. DNA methylation is often observed in chronic inflammatory diseases such as cholangitis, inflammatory bowel disease, hepatitis, and liver fibrosis. Studies have pointed out that at the same time, the abnormal expression of DNMTs associated with HBV persistent infection shows that abnormal gene promoter methylation exists in patients with CHB [53]. Studies have pointed out that OS can lead to epigenetic changes, which may be associated with the occurrence and development of CHB. Now there is no research on *GPX4* promoter methylation status in PBMCs from CHB patients and its relationship with immune tolerance.

In this study, we reported that CHB patients had lower GPX4 expression and higher methylation levels in PBMCs and serum than HCs. And, among CHB patients, those in the immune tolerance phase had lower GPX4 expression levels and higher methylation levels in PBMCs or serum than those in the immune activation phase and reactivation phase. The results indicate that there is a decrease in GPX4 expression in PBMCs of CHB patients caused by hypermethylation, which is most significant in the immune tolerance phase among all clinical stages, suggesting that this phenomenon may be involved in the development of immune tolerance leading to HBV chronic infection in CHB. In addition, the patients in the immune tolerance phase and inactive low replication phase had lower *STING* mRNA expression levels in PBMCs and lower serum levels of IFN-β than those in the immune activation phase and reactivation phase, and there was no significant difference with HCs, which suggests that STING may be involved in mediating the immune process of PBMCs against HBV, and may be a key target for breaking immune tolerance after HBV infection. However, the corresponding antiviral response was not stimulated by the high viral load in the immune tolerance phase, and the levels of STING and IFN-β were similar to those in healthy people. It has been found that the cellular redox homeostasis maintained by GPX4 is required for STING activation. GPX4 deficiency enhances cellular lipid peroxidation, thereby specifically inhibiting the cGAS-STING pathway [16]. Therefore, we hypothesized that the lower expression levels of STING and IFN-β in the immune tolerance phase may be associated with the lower expression of GPX4. Therefore, we analyzed the correlation of *GPX4* mRNA expression level and methylation level with *STING* mRNA expression level in PBMCs and serum IFN-β level in CHB patients. The results showed that *GPX4* mRNA level was significantly positively correlated with *STING* mRNA expression level in PBMCs and serum IFN-β level, and *GPX4* methylation level was significantly negatively correlated with *STING* mRNA expression level in PBMCs and serum IFN-β level. This supports our speculation. Then, we detected the OS markers in the peripheral blood serum of CHB patients and analyzed the correlation between them and *GPX4* promoter methylation level. The results showed that *GPX4* promoter methylation level was significantly negatively correlated with serum SOD level and significantly positively correlated with serum ROS and MDA levels, which was consistent with OS caused by GPX4 inhibition. Next, we analyzed the correlation between serum levels of inflammatory factors and *GPX4* promoter methylation levels in the peripheral blood of CHB patients. The results indicated that *GPX4* promoter methylation level was significantly positively correlated with serum TNF-α, IL-1β, and IL-6 levels, consistent with the role of GPX4 in inhibiting inflammation. Meanwhile, we analyzed the relationship between *GPX4* promoter methylation level in PBMCs and the viral load of CHB patients and found a clear positive correlation between them. So we speculate that HBV infection may inhibit the expression of *GPX4*, but further studies are needed to verify this hypothesis. These results support the hypothesis that GPX4 plays an important role in the pathogenesis and immune tolerance of CHB. In conclusion, we have reasons to assume that the high methylation level of the *GPX4* promoter plays an important role in the pathogenesis and immune tolerance of CHB, inhibits the antiviral effect of STING, and promotes the development of immune tolerance in CHB patients. Understanding this function of GPX4 may help DNMT inhibitors combined with various antioxidants and STING agonists to treat CHB, and improve the antiviral effect, to achieve the functional cure of CHB. However, it is noteworthy that the methylation status of the *GPX4* promoter was only detected in PBMCs and not in liver tissue, mainly because of the difficulty in obtaining liver tissue specimens from CHB patients. In addition, PBMCs, including monocytes, T cells, B cells, and NK cells, are considered to be the first line of defense of the immune system against inflammation, and previous studies have reported that various diseases can affect gene expression of PBMCs through host immunity or inflammatory response.

Our study has some limitations. First, the sample size was relatively small and all patients were from a single center, which may have led to selection bias. In addition, the possibility of false positive clinicopathological features cannot be ignored. Second, we only evaluated the correlation between GPX4 and the expression levels of related cytokines, and the specific regulatory relationship between them is unknown. In conclusion, the *GPX4* promoter was hypermethylated in CHB patients, especially in the immune tolerance phase. The methylation level of the *GPX4* promoter is correlated with the expression levels of STING, IFN-β, and related cytokines, suggesting that GPX4 may play an important role in the pathogenesis and immune tolerance of CHB, which may provide new ideas for achieving functional cure of CHB. But the further studies are needed to clarify the exact role of GPX4 in CHB pathogenesis and its potential as a therapeutic target.

### Electronic supplementary material

Below is the link to the electronic supplementary material.

Supplementary Material 1

## Data Availability

No datasets were generated or analysed during the current study.

## Abbreviations

HBV: Hepatitis B virus
GPX4: Glutathione peroxidase 4
CHB: Chronic hepatitis B
PBMCs: Peripheral blood mononuclear cells
HCs: Healthy controls
HCC: Hepatocellular carcinoma
WHO: World Health Organization
HBsAg: Hepatitis B surface antigen
HBeAg: Hepatitis B e antigen
HBV DNA: Hepatitis B DNA
ALT: Alanine aminotransferase
IFN: Interferon
ISGs: Interferon-stimulating genes
cGAS: Cyclic guanosine monophosphate–adenosine monophosphate synthase; cGAMP:Cyclic GMP-AMP
STING: Stimulator of interferon genes
HIV: Human immunodeficiency virus
HSV-1: Herpes simplex virus 1
HPV: Human papillomavirus
EBV: Epstein-Barr virus
ER: Endoplasmic reticulum
TBK1: TANK-binding kinase 1
IRF3: Interferon regulatory factor 3
NF-κB: Nuclear factor-κB
IL-1β: Interleukin-1β
IL-6: Interleukin-6
TNF-α: Tumor necrosis factor-α
MDA: Malonaldehyde
ROS: Reactive oxygen species
SOD: Superoxide dismutase
APC: Antigen-presenting cells
PMR: Percentage of the methylation reference value
GSH: Glutathione
DNMTs: DNA methyltransferases
IT: Immune tolerance phase
IA: Immune activation phase
LR: Inactive low replication phasee
RA: Reactivation phase
AFP: α-fetoprotein
AKP: Alkaline phosphatase
ALB: Albumin
AST: Aspartate aminotransferase
Cr: Creatinine
GGT: γ-glutamyl transferase
INR: International normalized ratio
NA: Not available
PLT: Platelet
PTA: Prothrombin activity
TBIL: Total bilirubin
