# Supplementary figures and images for: Hypermethylation of the glutathione peroxidase 4 gene promoter is associated with the occurrence of immune tolerance phase in chronic hepatitis B
Source: Virol J. 2024 Mar 21;21:72. doi: 10.1186/s12985-024-02346-6 (PMC10958902; doi:10.1186/s12985-024-02346-6)

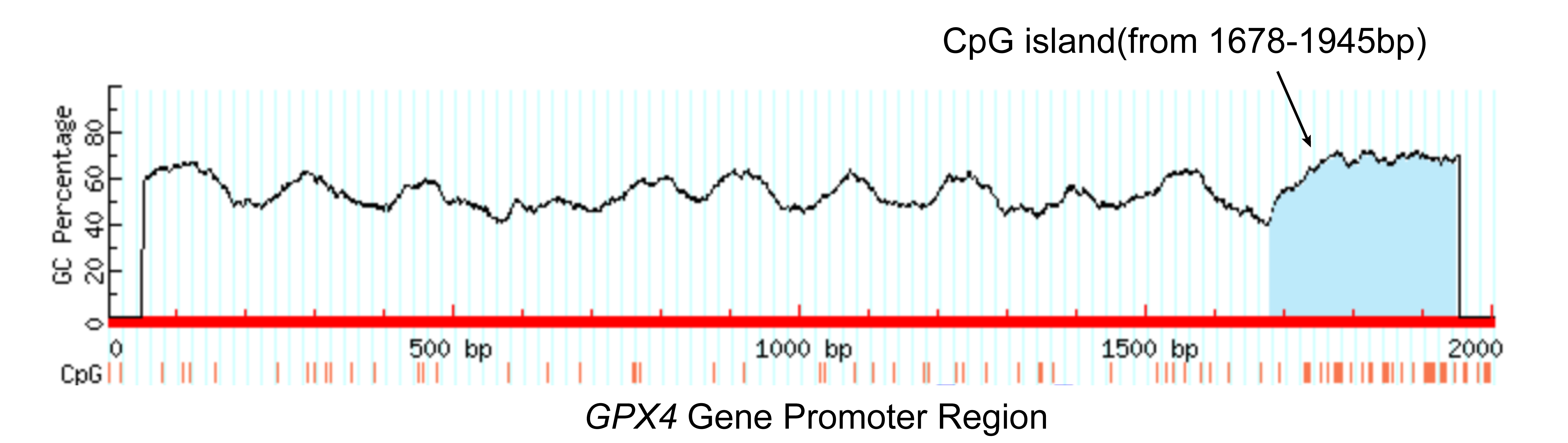

Supplement: Supplementary file 1 — Supplementary Material 1 [file 12985_2024_2346_MOESM1_ESM.tif]
